# Reconstruction and computational analysis of the microRNA regulation gene network in wheat drought response mechanisms

**DOI:** 10.18699/vjgb-24-98

**Published:** 2024-12

**Authors:** M.A. Kleshchev, A.V. Maltseva, E.A. Antropova, P.S. Demenkov, T.V. Ivanisenko, Y.L. Orlov, H. Chao, M. Chen, N.A . Kolchanov, V.A. Ivanisenko

**Affiliations:** Institute of Cytology and Genetics of the Siberian Branch of the Russian Academy of Sciences, Novosibirsk, Russia Research Center in the Field of Artificial Intelligence of Novosibirsk State University, Novosibirsk, Russia; Institute of Cytology and Genetics of the Siberian Branch of the Russian Academy of Sciences, Novosibirsk, Russia Novosibirsk State University, Novosibirsk, Russia Research Center in the Field of Artificial Intelligence of Novosibirsk State University, Novosibirsk, Russia; Institute of Cytology and Genetics of the Siberian Branch of the Russian Academy of Sciences, Novosibirsk, Russia Research Center in the Field of Artificial Intelligence of Novosibirsk State University, Novosibirsk, Russia; Institute of Cytology and Genetics of the Siberian Branch of the Russian Academy of Sciences, Novosibirsk, Russia Kurchatov Genomic Center of ICG SB RAS, Novosibirsk, Russia Novosibirsk State University, Novosibirsk, Russia Research Center in the Field of Artificial Intelligence of Novosibirsk State University, Novosibirsk, Russia; Institute of Cytology and Genetics of the Siberian Branch of the Russian Academy of Sciences, Novosibirsk, Russia Kurchatov Genomic Center of ICG SB RAS, Novosibirsk, Russia Novosibirsk State University, Novosibirsk, Russia Research Center in the Field of Artificial Intelligence of Novosibirsk State University, Novosibirsk, Russia; Agrarian and Technological Institute, Peoples’ Friendship University of Russia named after Patrice Lumumba, Moscow, Russia Digital Health Center, I.M. Sechenov First Moscow State Medical University of the Ministry of Health of the Russian Federation (Sechenov University), Moscow, Russia; Department of Bioinformatics, College of Life Sciences, Zhejiang University, Hangzhou, China; Department of Bioinformatics, College of Life Sciences, Zhejiang University, Hangzhou, China; Institute of Cytology and Genetics of the Siberian Branch of the Russian Academy of Sciences, Novosibirsk, Russia Kurchatov Genomic Center of ICG SB RAS, Novosibirsk, Russia Novosibirsk State University, Novosibirsk, Russia; Institute of Cytology and Genetics of the Siberian Branch of the Russian Academy of Sciences, Novosibirsk, Russia Kurchatov Genomic Center of ICG SB RAS, Novosibirsk, Russia Novosibirsk State University, Novosibirsk, Russia Research Center in the Field of Artificial Intelligence of Novosibirsk State University, Novosibirsk, Russia

**Keywords:** microRNA, bread wheat, drought, genes, genetic regulation, associative gene networks, plant bioinformatics, Smart crop knowledge base, ANDSystem computer tool, микроРНК, мягкая пшеница, дефицит влаги, гены, генетическая регуляция, ассоциативные генные сети, биоинформатика растений, база знаний Smart сrop, программно-информационная система ANDSystem

## Abstract

Drought is a critical factor limiting the productivity of bread wheat (Triticum aestivum L.), one of the key agricultural crops. Wheat adaptation to water deficit is ensured by complex molecular genetic mechanisms, including the coordinated work of multiple genes regulated by transcription factors and signaling non-coding RNAs, particularly microRNAs (miRNAs). miRNA-mediated regulation of gene expression is considered one of the main mechanisms of plant resistance to abiotic stresses. Studying these mechanisms necessitates computational systems biology methods. This work aims to reconstruct and analyze the gene network associated with miRNA regulation of wheat adaptation to drought. Using the ANDSystem software and the specialized Smart crop knowledge base adapted for wheat genetics and breeding, we reconstructed a wheat gene network responding to water deficit, comprising 144 genes, 1,017 proteins, and 21 wheat miRNAs. Analysis revealed that miRNAs primarily regulate genes controlling the morphogenesis of shoots and roots, crucial for morphological adaptation to drought. The key network components regulated by miRNAs are the MYBa and WRKY41 family transcription factors, heat-shock protein HSP90, and the RPM1 protein. These proteins are associated with phytohormone signaling pathways and calcium-dependent protein kinases significant in plant water deficit adaptation. Several miRNAs (MIR7757, MIR9653a, MIR9671 and MIR9672b) were identified that had not been previously discussed in wheat drought adaptation. These miRNAs regulate many network nodes and are promising candidates for experimental studies to enhance wheat resistance to water deficiency. The results obtained can find application in breeding for the development of new wheat varieties with increased resistance to water deficit, which is of substantial importance for agriculture in the context of climate change.

## Abstract (ru)

Недостаток влаги – критический фактор, ограничивающий продуктивность мягкой пшеницы (Triticum aestivum L.), одной из ключевых сельскохозяйственных культур. Адаптация пшеницы к водному дефициту обеспечивается комплексными молекулярно-генетическими механизмами, включающими согласованную работу множества генов, регулируемых транскрипционными факторами и сигнальными некодирующими РНК, в частности микроРНК. микроРНК – опосредованная регуляция экспрессии генов – рассматривается как один из основных механизмов устойчивости растений к абиотическим стрессам. Изучение этих сложных молекулярно-генетических механизмов требует применения методов компьютерной системной биологии. Цель данной работы – реконструкция и компьютерный анализ генной сети, связанной с микроРНК-регуляцией адаптации мягкой пшеницы к условиям недостаточного увлажнения. Для достижения этой цели использованы программно-информационная система ANDSystem и специализированная база знаний Smart crop, адаптированная для области генетики и селекции пшеницы. Нами была реконструирована генная сеть ответа пшеницы на водный дефицит, включающая 144 гена, 1017 белков и 21 микроРНК пшеницы. Анализ сети выявил, что микроРНК преимущественно регулируют гены, контролирующие процессы морфогенеза побегов и корней растений, что играет важную роль в морфологических адаптациях к засухе. Ключевыми компонентами генной сети, регулируемыми микроРНК, оказались транскрипционные факторы семейств MYB и WRKY, а также белок теплового шока HSP90 и белок RPM1. Эти белки связаны с сигнальными путями фитогормонов и кальций-зависимыми протеинкиназами, играющими существенную роль в адаптации растений к водному дефициту. Было идентифицировано несколько микроРНК (MIR7757, MIR9653a, MIR9671, MIR9672b), ранее не обсуждавшихся в контексте адаптации пшеницы к засухе, которые являются кандидатами для дальнейших экспериментальных исследований, направленных на усиление устойчивости пшеницы к недостатку влаги. Полученные результаты могут быть полезными для создания новых сортов пшеницы с повышенной устойчивостью к водному дефициту, что имеет существенное значение для сельского хозяйства в условиях изменения климата.

## Introduction

The productivity of bread wheat (Triticum aestivum L.) – a crucial agricultural crop – depends on many environmental factors, including micronutrient availability, temperature, moisture, and soil salinity. Water deficiency is the most important factor limiting wheat productivity (Pakul et al., 2018; Jeyasri et al., 2021). Therefore, studying the physiological and molecular genetic mechanisms of wheat adaptation to water deficiency is an urgent task, the solution of which is necessary for developing new drought-resistant varieties (Langridge, Reynolds, 2021) and improving agricultural technologies.

Plant resistance to insufficient moisture conditions is ensured by several physiological and morphological adaptations, which include enhanced apical growth and inhibition of lateral root growth, leaf abscission, changes in development rate, maintenance of tissue osmotic pressure, reduced transpiration through changes in stomatal apparatus functioning, and activation of cellular antioxidant defense. The functioning of these physiological mechanisms is provided by the coordinated work of numerous genes. It has been shown that water deficiency causes changes in the expression of genes activated by abscisic acid, genes encoding glutathione S-transferase (GST), and the dehydrin protein family (Ferdous et al., 2015).

Signal perception by receptors on the cell wall and cell membrane leads to the activation of intracellular signaling cascades, mainly due to increased levels of reactive oxygen species (ROS) and changes in calcium ion levels. Additionally, important mediators coordinating the initiation of signaling cascades are phytohormones such as abscisic acid (ABA), jasmonic acid (JA), salicylic acid (SA), and ethylene (ET). Stress-activated signaling cascades include, in particular, mitogen-activated protein kinase (MAPK) and calcium-dependent protein kinase (CDPK) signaling pathways. Kinases and phosphatases activate or suppress various transcription factors, which in turn regulate the activity of genes controlling adaptation to adverse conditions (Baillo et al., 2019).

Currently, five gene families are known to encode transcription factors regulating adaptation processes to water deficiency: bZIP (mainly AREB/ABF), DREB (AP2/EREBP), MYB/MYC, NAC, and WRKY (Gahlaut et al., 2016). Literature analysis shows that modification of these transcription factors through genetic engineering methods can enhance plant resistance to adverse environmental factors. For example, transgenic wheat plants containing the Arabidopsis (Arabidopsis thaliana) DREB1A gene showed increased resistance to drought and salt stress without yield reduction (Pellegrineschi et al., 2004). C.F. Niu and colleagues (2012) obtained transgenic wheat plants with increased expression of the TaWRKY2 and TaWRKY19 genes. These plants demonstrated improved resistance to drought and oxidative stress

Besides transcription factors, gene expression can also be regulated by signaling non-coding RNA molecules. These include circular RNAs (circRNAs), as well as linear long non-coding RNAs (lncRNAs) and microRNAs (Li N. et al., 2022). These signaling molecules can regulate the expression of any genes involved in stress response, including transcription factors, and the expression of genes encoding signaling RNAs can also change in response to stress, providing an additional level of regulation.

MicroRNAs are single-stranded non-coding RNA molecules 20–25 nucleotides in length that regulate gene activity in plants by binding to the target gene’s messenger RNA, leading to its degradation and translation inhibition (Ma, Hu, 2023). It has been revealed that microRNA expression changes in plants in response to water deficiency, which has been shown for many plant species, including wheat. In Triticeae species under drought conditions, the expression of microRNA genes miR159, miR1137, miR1318, miR168, and others changed, with the direction of expression changes depending on tissue type, plant developmental stage, and the duration and intensity of exposure (Alptekin et al., 2017). In response to water deficiency in wheat root tissues, there were changes in the expression of microRNA miR1119, its target – transcription factor MYC2, as well as changes in the expression of numerous stress-response genes, increased abscisic acid content, and cellular antioxidant system activity (Shamloo-Dashtpagerdi et al., 2023).

Thus, microRNA impact on transcription factors can lead to activity changes in entire gene sets. Therefore, microRNAs can be considered master regulators of gene networks that form regulatory modules together with transcription factors and their target genes, including those ensuring plant adaptation to abiotic stress (Zhang et al., 2022) and plant growth and development (Liebsch, Palatnik, 2020). Consequently, targeting microRNAs and their regulatory module activity could become a tool for genetic manipulation of agricultural crops to achieve optimal growth and development parameters (Wang H., Wang H., 2015).

Bioinformatic methods for integrating and analyzing large omics data, including gene network reconstruction methods, are particularly important in marker-assisted breeding (Chao et al., 2023). Bioinformatic analysis of gene networks can help identify regulatory modules involved in plant adaptation to adverse environmental factors and understand its molecular mechanisms.

Previously, the ANDSystem software and information system was developed for reconstructing gene networks based on information obtained from factographic databases and collected through automatic analysis of scientific publication texts (Ivanisenko V.A. et al., 2015, 2019; Ivanisenko T.V. et al., 2020, 2022). ANDSystem has been applied to solve problems in various areas of biology and biomedicine, including research on molecular genetic mechanisms of asthma development (Bragina et al., 2014; Saik et al., 2018; Zolotareva et al., 2019), lymphedema (Saik et al., 2019), tuberculosis (Bragina et al., 2016), hepatitis C (Saik et al., 2016), coronavirus infection (Ivanisenko V.A. et al., 2022), Huntington’s disease (Bragina et al., 2023), glioma (Rogachev et al., 2021), post-operative delirium (Ivanisenko V.A. et al., 2023), hepatocellular carcinoma (Antropova et al., 2023), and study of the proteomic profile of cosmonauts (Larina et al., 2015; Pastushkova et al., 2019).

In the field of plant biology, ANDSystem has been used for reconstruction and analysis of the regulatory gene network of cell wall functioning in A. thaliana L. leaves in response to insufficient moisture (Volyanskaya et al., 2023). Based on ANDSystem, the SOLANUM TUBEROSUM knowledge base was created, containing information about genetic regulation of potato metabolic pathways (Ivanisenko T.V. et al., 2018), and prioritization of potato genes involved in the formation of agronomically valuable plant traits was conducted (Demenkov et al., 2019). It should also be noted that the ANDSystem software and information system was previously used for reconstructing gene networks describing microRNA regulation of the external apoptosis pathway (Khlebodarova et al., 2023).

The aim of this work is to reconstruct and analyze the gene network that regulates wheat adaptation to insufficient moisture conditions through microRNAs.

## Materials and methods

Search for information about drought response genes. Information about bread wheat genes experimentally proven to be associated with plant adaptation to drought conditions was extracted from full-text experimental and review articles indexed in PubMed (https://pubmed.ncbi.nlm.nih.gov/) as of September 2024. The search was conducted using keywords “wheat”, “Triticum aestivum”, “drought”, “drought tolerance”, “gene”, “genetic”, “regulation” and their combinations.

Additionally, information about genes related to water deficit response was extracted from the AmiGO gene ontology database for the term “response to water deprivation” (term ID GO:0009414). Furthermore, genes associated with the term “response to water deficiency” in the ANDSystem software and information system were included in the list of drought response genes. As a result, a list of genes shown to be involved in wheat adaptation to water deficit was compiled. This list was used as input data for gene network reconstruction.

Smart crop Knowledge Base. This work utilized the Smart crop knowledge base, which is a specialized version of the ANDSystem software and information system focused on rice and wheat genetics and breeding. Three key modules of ANDSystem were customized for the subject area:

Domain-specific ontology module. This module contains expanded dictionaries covering various research objects, such as genes, proteins, metabolites, non-coding RNAs/ microRNAs, biological processes, genetic biomarkers, QTL polymorphisms, plant varieties, breeding-significant qualities, phenotypic traits, diseases, pathogens, pests, resistance markers to plant protection products, molecular targets for chemical plant protection products, biotic and abiotic factors, plant protection products (herbicides), and others. Various databases and ontologies were used to form the dictionaries, including NCBI Gene, ChEBI, MirBase, Gene Ontology, Wheat Ontology, Rice Ontology, Wheat Trait and Phenotype Ontology, The International Herbicide-Resistant Weed Database, and others. The dictionaries were supplemented with synonyms and spelling variants of the names to improve object recognition in texts.

Information extraction module from factographic databases.This module performs automated data extraction from various sources, including relational databases (e. g., ChEBI), ontologies in OBO and OWL formats (using the ROBOT tool), text files in tabular formats (CSV, TSV), and PSI-MI XML 2.5 formats. Specialized extractor programs were created to process information from databases such as NCBI Gene, ToppGene, GrainGenes, IntAc, and others.

Text mining module using semantic linguistic templates. This module is designed to extract knowledge from text sources (scientific articles, patents) using semantic linguistic templates

The development of new templates and adaptation of existing ones in ANDSystem allowed for effective identification and extraction of various types of interactions between objects. The templates cover such interaction types as associations, regulation of gene and protein expression and activity, physical interactions, catalytic reactions, participation in biological processes, marker relationships, and others. In total, more than 2,000 templates were developed and used, significantly improving the accuracy and completeness of information extraction.

Customizing ANDSystem for the field of rice and wheat breeding and genetics allowed for the integration of data from various sources and ensured effective extraction and analysis of knowledge necessary for research in this subject area.

**Gene network reconstruction and analysis. **Gene network reconstruction and analysis were performed using the Query Master of the ANDVisio software module (Demenkov et al., 2012), which serves as the user interface in the ANDSystem and Smart crop systems.

Proteins and genes important for the reconstructed gene network functioning were identified using the “NetworkConnectivity” indicator, which characterizes the number of connections between a given network node and other nodes. Genes and proteins were then ranked according to this indicator to find the most significant nodes in the network. Functional annotation of the gene set (analysis of overrepresentation of Gene Ontology terms and KEGG pathways) represented in the network was conducted using the Database for Annotation, Visualization and Integrated Discovery (DAVID ver-sion 2021; https://david.ncifcrf.gov/) with default settings (statistical significance was considered at p-value < 0.05 with Bonferroni correction).

## Results and discussion

**Fig. 1.**
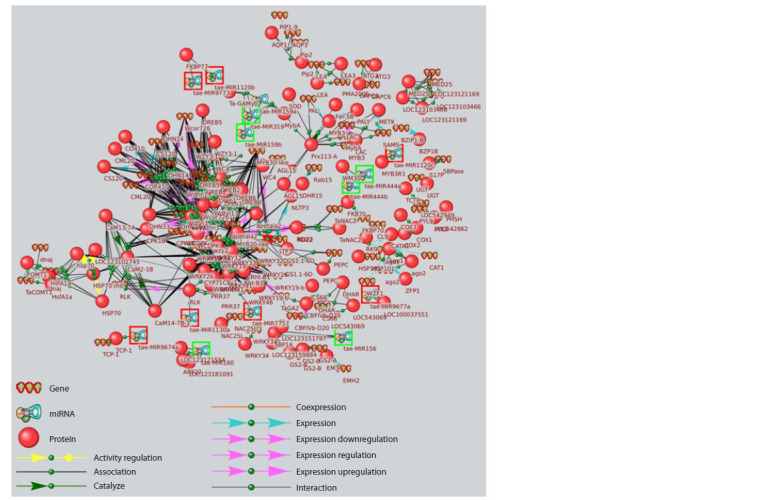
Associative network of genes and proteins experimentally proven to be involved in wheat adaptation to moisture deficit, supplemented with microRNAs directly regulating them. Green frames indicate microRNAs with data linking them to drought, red frames indicate microRNAs without such data.

Analysis of published literature (Nagy et al., 2013; Gupta et al., 2014; Liu et al., 2015; Gahlaut et al., 2016; Shojaee et al., 2022) revealed 130 genes involved in wheat adaptation to moisture deficiency. Additionally, 15 genes were associated with the Gene Ontology term “response to water deprivation” (term ID GO:0009414). Further, using the Smart crop knowledge base of the ANDSystem software and information system, 59 genes involved in wheat adaptation to moisture deficiency were discovered. The resulting list of 204 genes shown to be involved in wheat adaptation to insufficient moisture (drought response genes) is provided in Supplementary Material 11. Using this gene list as input data, we reconstructed an associative gene network, to which we added microRNAs that, according to the Smart crop knowledge base, directly regulate at least one network component. This associative network (Fig. 1) included 75 genes, 98 proteins, and 14 wheat microRNAs, as well as 695 interactions between network components. Of these, the following connection types were represented: 594 connections – “association”, 39 – “expression regulation”, 21 – “interaction”, 18 – “expression”, 12 – “activity regulation”, 7 – “catalysis”, 2 – “expression enhancement”, and 1 connection each for “expression suppression” and “coexpression” types. The list of microRNAs and their target genes included in the drought response network, established according to Smart crop data, is shown in Table 1.


Supplementary Materials are available in the online version of the paper: https://vavilov.elpub.ru/jour/manager/files/Suppl_Kleshchev_Engl_28_8.xlsx


**Table 1.**
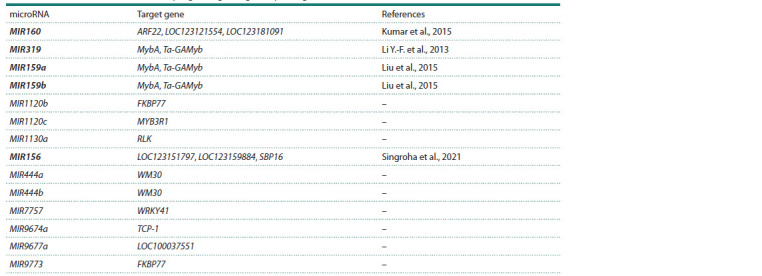
List of wheat microRNAs directly regulating drought response genes Notе. microRNAs that have been experimentally shown to change expression in response to moisture deficiency are highlighted in bold. References to the corresponding literature sources are provided for these microRNAs

These microRNAs primarily target genes encoding transcription factors from the GAMYB (MybA, Ta-GAMyb, MYB3R1), WRKY (WRKY41) families, auxin response factor (ARF22, LOC123121554, LOC123181091), MADS-box transcription factor (WM30), and SQUAMOSA family transcription factor (LOC123151797, LOC123159884, SBP16).

GAMYB transcription factors, which have highly conserved binding sites with MIR159a (Millar et al., 2019), participate in gibberellin-mediated activation of hydrolase gene expression in the seed aleurone layer (Woodger et al., 2003). In vegetative plant parts, MIR159 suppresses the expression of GAMYB transcription factor, which is a growth inhibitor ensuring normal plant development (Millar et al., 2019). MIR159 expression changes in response to drought, along with changes in GAMyb gene expression in potato (Yang J. et al., 2014) and bread wheat (Liu et al., 2015). Additionally, the MybA gene product regulates peroxidase gene expression (Wei et al., 2021), contributing to plant adaptation to adverse environmental factors. MIR160 targets genes encoding ARF transcription factor, a key component ensuring plant response to auxins (Li Y. et al., 2023) – phytohormones that, in particular, stimulate apical dominance, promoting root length growth, which is a morphological adaptation of plants to moisture deficiency.

Besides transcription factors, another microRNA target in the drought response gene network is the RLK serine/threonine kinase gene, which interacts with calmodulins and participates in plant adaptation to abiotic stress (Virdi et al., 2015).

Thus, analysis of the gene network, which includes genes and proteins, the role of which in drought response has been experimentally shown, identified several microRNAs regulating important nodes of this gene network (transcription factors), with some microRNAs (MIR1120, MIR1120c, MIR1130a, MIR444a, MIR444b, MIR7757, MIR9674a, MIR9677a, MIR9773) not having been previously discussed in literature in connection with wheat adaptation to drought, which may be promising for further research.

However, it should be noted that microRNAs often have many target genes, which may also be components of the drought response gene network, although their role is not currently experimentally established. Additionally, microRNAs can regulate genes controlling stress response not only directly but also through intermediaries. Therefore, using the Smart crop knowledge base, the initial gene network was supplemented with the following components: 1) all predicted, according to Smart crop data, targets of those 14 microRNAs that directly regulate known drought response genes and are listed in Table 1; 2) genes and proteins directly connected to drought response genes, as well as their regulating microRNAs.

**Table 2.**
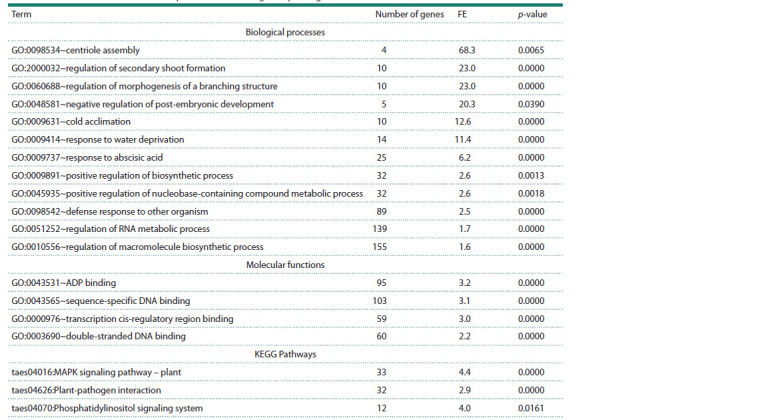
Functional annotation of the expanded wheat drought response gene network Notе. FE – fold enrichment; p-value – statistical significance indicator of gene and protein enrichment in the associative network with Bonferroni correction.

The resulting associative network is presented in Supplementary Material 2. The list of genes and proteins included in this network is provided in Supplementary Material 3. The network includes 144 genes, 1,017 proteins, and 21 wheat microRNAs, as well as 5,188 connections between network components. Of these, 4,158 connections correspond to the “association” type, 372 connections, to “interaction”, 329 connections, to “catalysis”, 180 connections, to “expression regulation”, 42 connections, to “activity regulation”, 24 connections, to “cleavage”, 21 connections, to “expression”, 15 connections, to “expression suppression”, 12 connections, to “expression enhancement”, and 7 connections, to “coexpression”.

Functional annotation of all components (genes and proteins) of the expanded associative gene network is shown in Table 2. As seen from Table 2, gene network components are significantly enriched with terms characterizing biological processes related to centriole assembly, shoot morphogenesis (regulation of morphogenesis of a branching structure), delayed post-embryonic development, response to abiotic and biotic stress factors, and response to abscisic acid. Additionally, gene network components are involved in mitogendependent protein kinase and phosphatidylinositol signaling pathways.

Interestingly, the expanded gene network includes genes involved not only in adaptation to water deficit (Gene Ontology term GO:0009414, “response to water deprivation”) but also in plant response to other adverse factors, including cold adaptation (Gene Ontology term GO:0009631, “cold acclimation”) and interaction with pathogens (KEGG pathway taes04626, “plant-pathogen interaction”). This is likely due to the fact that products of the same genes can participate in plant response to various stress factors, ensuring plant adaptation to a complex of adverse factors. In particular, genes in our gene network associated with the term “cold acclimation” (GO:0009631) belong to the families of dehydrins and cold-shock proteins.

It is known that proteins of the dehydrin family, by participating in cell membrane stabilization, contribute to plant adaptation to various abiotic stress factors, including moisture deficiency, temperature reduction, and soil salinity (Szlachtowska, Rurek, 2023). On the other hand, cold-shock proteins, which are crucial participants in plant cold adaptation, can also play a certain role in plant response to moisture deficit by regulating the activity of genes, the products of which participate in cellular antioxidant defense (Yu T.F. et al., 2017; Li C. et al., 2021a). Additionally, according to literature, such components of the drought response gene network as calmodulins (Cheval et al., 2013) and WRKY transcription factors (Wani et al., 2021) can also participate in regulating plant immunity and protecting plants from pathogens.

In the expanded associative gene network, the highest number of connections with other network components (Network Connectivity) was found for MYB30-like transcription factor, calmodulin proteins (CaM13-7A, CaM14-7B-1, CaM2- 1B), APETALA2-like protein, which is a member of the APETALA2 (AP2) subfamily of AP2/Ethylene Responsive Factor (ERF) transcription factors, as well as RHT1 protein, WRKY41 transcription factor, and cytochrome P450 (CYP71C8v1). Genes encoding these proteins have already been discussed in literature as controlling plant response to moisture deficit

MYB transcription factors are among the most common families of transcription factors in plants that participate in plant development and response to various adverse environmental factors, including moisture deficiency. MYB transcription factors, by binding to MYB cis-elements in promoters of multiple target genes, regulate a number of biological processes, particularly flavonoid biosynthesis, which is necessary for protection against oxidative stress. Additionally, MYB transcription factors activate genes controlling epicuticular wax formation, which reduces moisture evaporation from plant leaves (Wang X. et al., 2021).

It is known that calcium is a crucial secondary messenger, the concentration of which changes in response to various adverse factors, including moisture deficiency. Calmodulins and calmodulin-like proteins, by binding to calcium ions, change their conformation and modulate the activity of numerous other proteins, including kinases, transcription factors, transporters, and enzymes of various metabolic pathways that ensure plant adaptation to the environment (Ranty et al., 2016). In particular, increased expression of a gene encoding one of the calmodulin family proteins in wheat was observed in response to moisture deficiency and increased salinity, and expression of this gene in transgenic Arabidopsis plants increased their resistance to these adverse factors (Li Y. et al., 2022).

Proteins of the APETALA2 (AP2) subfamily belong to the AP2/Ethylene Responsive Factor (ERF) family of transcription factors, which regulate the expression of genes providing adaptation to adverse environmental conditions, including drought (Park S.Y., Grabau, 2016; Srivastava, Ku- mar, 2018). Expression of genes encoding AP2 subfamily proteins, TaAP2- 1-1A, TaAP2-1-1D, was increased in response to drought in wheat (Yu Y. et al., 2022).

Cytochrome P450 family proteins are enzymes involved in multiple metabolic pathways for the synthesis of plant secondary metabolites, phytohormones, and antioxidants, which play an important role in plant adaptation to the environment (Pandian et al., 2020). In the study (Li Y., Wei, 2020), it was shown that in wheat, in response to drought, there were changes in the expression of 77 genes encoding cytochrome P450s, which participate in the biosynthesis of abscisic acid, an important mediator activating various signaling cascades in plant stress responses, as well as cytochrome P450s involved in the synthesis of flavonoids, which play an important role in plant cell antioxidant defense.

Among the intermediary proteins connected to experimentally found drought response genes, the gene LOC123186119, encoding the disease resistance protein RPM1, had the highest number of connections with other network components. It is connected to all WRKY family transcription factors represented in the network, as well as to calcium-dependent protein kinases 7 and 19. Additionally, the RPM1 protein is a target of microRNA MIR7757. The list of 21 microRNAs associated with components of the expanded gene network is shown in Table 3. The complete list of 984 predicted microRNA targets according to the Smart crop database is presented in Supplementary Material 4.

**Table 3.**
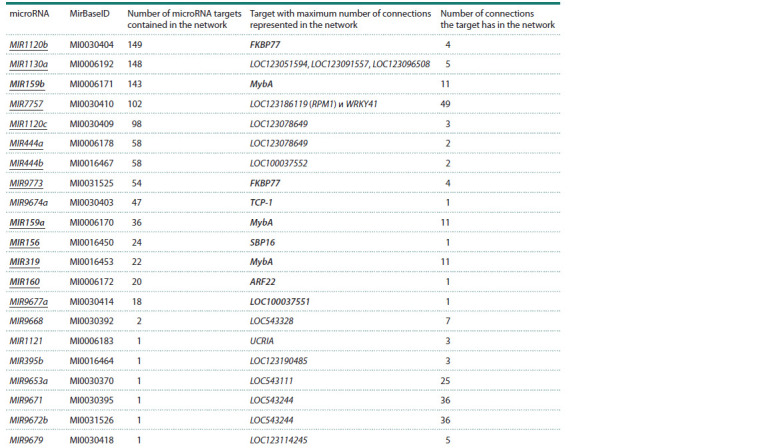
List of microRNAs and their target genes with the highest number of connections in the network Notе. MicroRNAs and target genes with known significance in drought adaptation in wheat are highlighted in bold. MicroRNAs that directly regulate known water deficit response genes are underlined.

The results of functional annotation of microRNA target genes in the associative network are shown in Table 4. As seen from Table 4, microRNA targets in the drought response gene network are involved in morphogenesis processes of plant lateral shoots and roots, as well as plant immunity, purine transport and metabolism, and transcription factor functioning. Genes controlling shoot morphogenesis processes in the expanded gene network (see Supplementary Material 2) mainly include targets of microRNA miR319, encoding the TEOSINTE BRANCHED/CYCLOIDEA/PCF (TCP) transcription factor family, which is involved in forming plant shoot and root architecture (Tokizawa et al., 2023), including root hair formation (Wang M.Y. et al., 2013), which is an important morphological adaptation of plants to moisture deficiency.

**Table 4.**
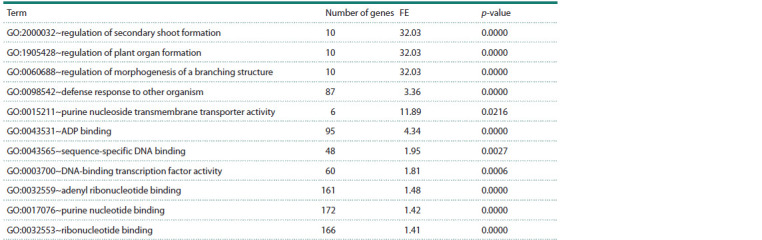
Functional annotation of wheat microRNA target genes in the drought response gene network Notе. FE – foldenrichment, p-value – statistical significance indicator of enrichment with Bonferroni correction.

The involvement of TEOSINTE BRANCHED/CYCLOIDEA/ PCF (TCP) family transcription factors in response to insufficient moisture is discussed in literature (Manna et al., 2021), although their participation in moisture deficit response has not been shown for wheat. Knockout of miR319 family members IbmiR319a and IbmiR319c in transgenic sweet potato plants led to increased sensitivity to moisture deficiency, increased number of stomata, decreased lignin content, and disruption of hormonal regulation of plant growth (Ren et al., 2022). The authors suggest that these morphological changes are caused by changes in the expression of transcription factor TCP11/17, which is a target of IbmiR319a and IbmiR319c.

Among the 21 microRNAs in the expanded gene network (see Table 3), 14 were directly connected to genes, the role of which in wheat adaptation to moisture deficiency has been experimentally proven. Seven microRNAs (MIR9668, MIR1121, MIR395b, MIR9653a, MIR9671, MIR9672b, MIR9679) were connected to drought response genes through an intermediary. MicroRNAs MIR1120b, MIR1130a, MIR159b, MIR7757 и MIR1120c had the highest number of connections with other network components

**Fig. 2.**
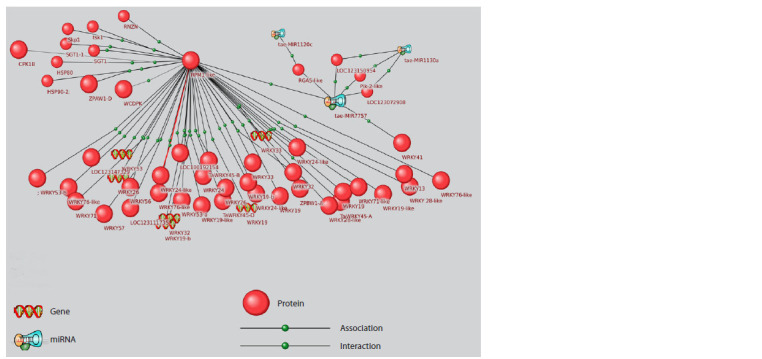
Associative network of microRNA MIR7757, its targets, and intermediaries connected to the targets Large spheres indicate proteins experimentally shown to be involved in drought response.

In particular, it is interesting to note that not only did microRNA MIR7757 have connections with many network nodes (102), but its target, the LOC123186119 gene encoding disease resistance protein RPM1, was connected to the highest number (49) of other network nodes. These nodes include a set of WRKY family transcription factors, as well as calcium-dependent protein kinases 7 (WCDPK) and 19 (CPK 1B), SKP1 and SGT1 proteins, and heat shock protein HSP80 (Fig. 2).

Numerous data obtained from different plant species indicate that WRKY family transcription factors play a crucial role in adaptation to various stress factors, including moisture deficit. Increased expression of WRKY transcription factors contributes to reduced ion loss, activation of leaf stomatal apparatus, decreased moisture loss, and reduced reactive oxygen species content (Khoso et al., 2022).

It is known that WRKY transcription factors modulate the activity of signaling pathways of phytohormones – salicylic acid, ethylene, abscisic acid, jasmonic acid, mitogen-activated protein kinase MAPK (Jiang et al., 2017), as well as calmodulins, including through physical interaction with the calcium domain in calmodulins (Park C.Y. et al., 2005). The activity of WRKY transcription factors is controlled by various signaling pathways and phytohormones, including ethylene (Li J. et al., 2006), abscisic acid (Chen et al., 2010), and MAPK signaling pathway (Mao et al., 2011), which ensures changes in WRKY activity depending on environmental conditions. Thus, WRKY transcription factors are a crucial regulatory link in plant stress response, affecting the activity of multiple genes regulating adaptation, while WRKY activity can change depending on the nature of the impact, providing flexible plant adaptation to changing environmental conditions.

Calmodulins and calcium-dependent protein kinases, by binding to calcium ions, the concentration of which increases in response to stress factors, change the functioning of abscisic acid signaling pathways, which in turn causes changes in seed maturation rate, stomatal closure, and reduced reactive oxygen species content (Asano et al., 2012).

The SKP1 protein is part of the SCF (Skp1-Cullin 1-F-box) complex, which is a ubiquitin ligase playing an important role in hormonal signal transmission, circadian rhythm regulation, plant growth and development (Hong et al., 2012), and adaptation to adverse factors (Saxena et al., 2023). Thus, MIR7757 may be a crucial master regulator of the moisture deficit response gene network, acting both directly on the WRKY41 transcription factor and through an intermediary – RPM1-like protein, coordinating phytohormone signaling pathways, MAPK, and calcium-dependent protein kinases. This protein plays an important role in plant immunity; however, its significance in wheat response to water deficit is unknown, although it was reported that PRM1 gene expression was increased in grape leaves in response to moisture deficiency (Haider et al., 2017). Additionally, there is no data on changes in wheat MIR7757 microRNA expression under moisture deficiency; therefore, this microRNA, as well as other microRNAs with a high number of network node connections and their target genes, are promising candidates for experimental investigation of microRNA regulation of wheat response to water deficit.

**Fig. 3.**
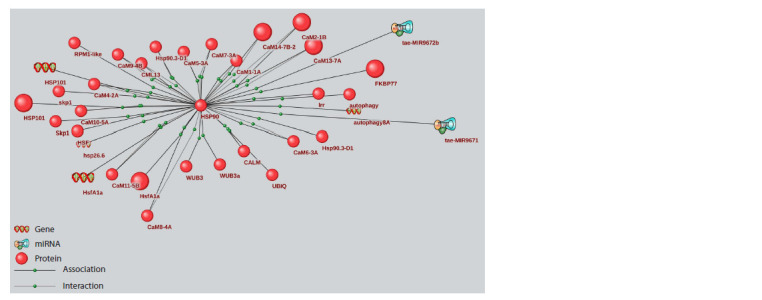
Associative network of microRNAs MIR9671, MIR9672b, their targets, and proteins connected to the targets Large spheres indicate proteins experimentally shown to be involved in drought response.

The target of two other microRNAs, MIR9671 and MIR9672b, heat shock protein 90, encoded by the LOC543244 gene, also has extensive (n = 36) connections with other gene network nodes, namely calmodulins (CaM14-7B-1, LOC123104984, etc.), heat shock protein 101, SKP1 and RPM1 proteins discussed above, heat stress transcription factor HSf1a, and polyubiquitin UBIQ (Fig. 3).

It is known that the HSP90 protein, a highly conserved chaperone, is a crucial component of eukaryotic cell homeostasis and participates in plant adaptation to various types of abiotic stress, modulation of plant growth and development by interacting with auxin and jasmonic acid signaling pathways. The HSP90 protein, together with its co-chaperones, stabilizes the auxin receptor complex under conditions of increased air temperature (an environmental factor that often accompanies moisture deficiency) and promotes physiological and morphological adaptations induced by auxin, particularly root elongation (di Donato, Geisler, 2019). Additionally, HSP90, by interacting with protein ligases, assists in the removal of damaged proteins.

It should be noted that numerous calmodulin proteins, by binding to calcium ions during stress, not only activate calcium-dependent protein kinase signaling pathways but also activate HSP90 expression (Virdi et al., 2011), providing additional heat shock protein-mediated activation of the plant hormonal system. Thus, microRNAs MIR9671 and MIR9672b, through their target HSP90, can modulate hormonal signaling of auxin and jasmonic acid, as well as the functioning of the protein ubiquitination system during abiotic stress.

Considering the important role of the HSP90 protein in response to abiotic stress, it can be hypothesized that enhancing its expression by artificially weakening the activity or expression of microRNAs MIR9671, MIR9672b may increase wheat plant resistance to moisture deficiency. However, it should be noted that HSP90 has a pleiotropic effect, affecting a significant number of cell signaling pathways (di Donato, Geisler, 2019), therefore microRNA-mediated weakening of its expression may be necessary for adaptive changes in some signaling pathways at a certain stage of plant development or during environmental changes.

Thus, microRNAs MIR9671, MIR9672b, along with MIR7757, which were not previously discussed in literature in connection with wheat response to drought, may be promising for further experimental investigation of microRNA regulation of bread wheat response to water deficit.

Several experiments conducted on various plant species have shown that artificial modulation of microRNA expression allows changing regulatory gene network functioning, affecting the expression of genes responsible for adaptation to adverse environmental conditions or the formation of certain economically valuable traits. Modern genetic engineering technologies – RNA interference, creation of special vectors expressing specific microRNAs, as well as genome editing methods such as CRISPR/Cas9 and Transcription activatorlike effector nucleases (TALEN) – make it possible to enhance or weaken microRNA expression and activity depending on whether the products of microRNA target genes have a stimulating or weakening effect on target biological processes (Abbas et al., 2022; Raza et al., 2023). For example, an artificial increase in miR319 expression using special vectors, as well as an artificial decrease in expression of its targets, TEOSINTE BRANCHED/CYCLOIDEA/PCF (TCP) transcription factors, led to increased cold resistance in rice plants (Yang C. et al., 2013). In another study (Ni et al., 2013), suppression of miR169 expression increased expression of its target – transcription factor GmNFYA3, which improved soybean plant drought resistance through changes in expression of genes related to water deficit adaptation. An artificial increase in miR172b-3p expression in transgenic potato led to weakened expression of the ERFRAP2-7-like gene and enhanced carbon fixation by plants (Raza et al., 2023).

Additionally, patents have been obtained for transgenic plants, created using microRNAs, that have increased productivity and resistance to adverse environmental factors, demonstrating successful practical use of microRNAs for creating new plant varieties. For example, transgenic rice with increased expression of Osa-miR393 microRNA and enhanced tillering was obtained (patent CN102533760A (Wang S., Zhang, 2011)). Transgenic tomato plants with suppressed expression of miR156e-3p microRNA and increased resistance to low temperatures were created (patent CN111705077B (Zhou et al., 2020)).

Thus, modulation of microRNA activity using genetic engineering may become a promising method of modern biotechnology aimed at increasing plant resistance to adverse environmental conditions, including moisture deficiency, and ultimately their productivity.

## Conclusion

Using the Smart crop knowledge base of the ANDSystem software and information system, reconstruction of the gene network of microRNA regulation of bread wheat adaptation to moisture deficiency was performed. Genes in the network regulate root and shoot morphogenesis processes, response to abiotic and biotic stress factors, and are involved in signaling pathways of abscisic acid and calcium-dependent protein kinases.

Twenty-one microRNAs regulating the wheat drought response gene network were identified, the targets of which are mainly involved in controlling plant morphogenesis processes. The most significant nodes in this network regulated by microRNAs are MYBa and WRKY41 transcription factors, HSP90 heat shock protein, and RPM1 protein, which is connected to WRKY family transcription factor proteins, calcium-dependent protein kinases, and phytohormone signaling pathways – auxin, jasmonic acid, and abscisic acid, which are crucial in controlling plant adaptation to moisture deficiency. Several microRNAs that were not previously discussed in literature in connection with drought adaptation (MIR7757, MIR9671, MIR9672b) regulate a significant number of network nodes and therefore may be promising for further experimental investigation of microRNA regulation of bread wheat response to water deficit.

## Conflict of interest

The authors declare no conflict of interest.
